# Supplementary material for: Shedding light on development: Leveraging the new nightlights data to measure economic progress
Source: PLoS One. 2025 Feb 3;20(2):e0318482. doi: 10.1371/journal.pone.0318482 (PMC11790135; doi:10.1371/journal.pone.0318482)
Supplement: S1 Appendix — (DOCX) [file pone.0318482.s001.docx]

**S1 Appendix: Harmonised Nightlights Data**

The DMSP NTL data is a time series dataset with a spatial resolution of 30 arc-seconds. However, the raw DMSP NTL data is not comparable across years. To overcome this limitation, a stepwise calibration approach was used [1] to generate a temporally consistent NTL dataset, which outperforms traditional approaches in terms of temporal trends and correlation with electricity consumption data. In contrast, the VIIRS Day/Night Band (DNB) data provides radiance records with improved radiometric resolution and a higher spatial resolution of 15 arc-seconds. The monthly VIIRS NTL data were further preprocessed and composited as annual time series data. The dataset spans from 2012 to 2020.

To generate the harmonized series from 1992 to 2020, a three-step framework was employed [1]. Firstly, annual VIIRS NTL data was produced from monthly observations and noise from temporary lights was excluded. Secondly, the relationship between processed VIIRS data and DMSP NTL data in 2013 was quantified using a sigmoid function. Thirdly, the derived relationship was applied globally to obtain DMSP-like data from VIIRS. The consistent NTL dataset was generated by integrating the temporally calibrated DMSP NTL data from 1992 to 2013 and DMSP-like NTL data from VIIRS from 2014 to 2020 [1].

**References**

1. Li X, Zhou Y, Zhao M, Zhao X. Harmonization of DMSP and VIIRS nighttime light data from 1992-2021 at the global scale. Figshare. Scientific Data. 2020;7:168. Available from: <https://doi.org/10.6084/m9.figshare.9828827.v5>
