# Supplementary material for: Shedding light on development: Leveraging the new nightlights data to measure economic progress
Source: PLoS One. 2025 Feb 3;20(2):e0318482. doi: 10.1371/journal.pone.0318482 (PMC11790135; doi:10.1371/journal.pone.0318482)
Supplement: S2 Appendix — (DOCX) [file pone.0318482.s002.docx]

**S2 Appendix: Literature Review**

A few previous studies in economics have assessed the accuracy of NTL data in predicting GDP at various levels, such as national, regional and sub-regional levels [1, 2, 3, 4] or to evaluate the accuracy of official national account statistics [5, 6]. Some studies have found that NTL can predict wealth indicators [7] as well as human development outcomes at the local level created from DHS data on education, health and wealth [8]. However, these studies have all used DMSP data. A parallel literature in remote sensing and artificial intelligence uses a combination of these alternative data sources and computer vision models to predict estimates of inequality or wealth.

For example, one study [9] trained a convolutional neural network (CNN) with high-resolution satellite imagery to predict local economic output for 5 countries using a transfer-learning approach. They did so by combining information from an image classification dataset trained to predict NTL corresponding to daytime imagery and information from the DHS and Living Standard Measurement Survey (LSMS). Since then, there have been several studies [10, 11] in this direction. Another study [12] adopted a similar approach but tested whether it could be used to measure changes in poverty over time. However, these methods do not come without limitations. According to a survey conducted by the Asian Development Bank (ADB) and the United Nations Economic and Social Commission for Asia and the Pacific, incorporating big data into their programs is a challenge for 7 out of 16 National Statistical Offices in the ADB member countries due to difficulties in accessing these alternative data sources [13]. Acquiring high-resolution satellite images is expensive, and when combined with the computer-intensive deep learning methods required in their analysis, widespread adoption becomes challenging. It is especially difficult for development organizations grappling with limited resources. At the same time, these deep learning models are considered “black-boxes” in the sense that it is difficult for policy makers to understand and apply them to solving real world problems [14, 15]. They are often criticized for attempting to only maximize model performance rather than model interpretability which is especially important when developing policies and interventions that have the potential of greatly affecting people’s well-being [16]. For instance, a recent review [17] of 32 papers on wealth/poverty prediction that used satellite images as one of their inputs and deep neural networks as their method to predict survey data, finds that almost all literature conducted so far does not meet the requirements for interpretability and explainability, things that are important for wider acceptance/adoption of the research in the development community. They establish specific criteria for interpretability and explainability in such studies. These include emphasizing understandable descriptions of the model’s properties, potentially using tools like heat maps for complex models to illustrate what the model reacts to.; and detailed explanations for predictions, addressing why certain areas are classified as poor or not and exploring what changes in the satellite data would be needed for an area to transition from being poor to not so poor. These criteria aim to enhance the acceptance and adoption of research findings, particularly within the development community. They note that almost all research conducted has been by the “technical community” and therefore domain knowledge is mostly missing and should be integrated in

future research. Further, model outputs from these studies are rarely made publicly available as rasters, data frames or other data products that would be easy to incorporate into studies of the influence of poverty and household income by non-experts in remote sensing.

**References**

1. Chen X, Nordhaus WD. Using luminosity data as a proxy for economic statistics. Proceedings of the National Academy of Sciences. 2011 May 24;108(21):8589-94. doi:10.1073/pnas.1017031108
2. Henderson JV, Storeygard A, Weil DN. Measuring economic growth from outer space. American economic review. 2012 Apr 1;102(2):994-1028. doi: 10.1257/aer.102.2.994
3. Keola S, Andersson M, Hall O. Monitoring economic development from space: using nighttime light and land cover data to measure economic growth. World Development. 2015 Feb 1;66:322-34. doi: 10.1016/j.worlddev.2014.08.017
4. Hodler R, Raschky PA. Regional favoritism. The Quarterly Journal of Economics. 2014 May 1;129(2):995-1033. doi: 10.1093/qje/qju004
5. Clark H, Pinkovskiy M, Sala-i-Martin X. China's GDP growth may be understated. China Economic Review. 2020 Aug 1;62:101243. doi:10.1016/j.chieco.2018.10.010
6. Pinkovskiy M, Sala-i-Martin X. Lights, camera… income! Illuminating the national accounts-household surveys debate. The Quarterly Journal of Economics. 2016 May 1;131(2):579-631. doi: 10.1093/qje/qjw003
7. Weidmann NB, Schutte S. Using night light emissions for the prediction of local wealth. Journal of Peace Research. 2017 Mar;54(2):125-40. doi: 10.1177/0022343316630359
8. Bruederle A, Hodler R. Nighttime lights as a proxy for human development at the local level. PloS one. 2018 Sep 5;13(9):e0202231. doi:10.1371/journal.pone.0202231
9. Jean N, Burke M, Xie M, Davis WM, Lobell DB, Ermon S. Combining satellite imagery and machine learning to predict poverty. Science. 2016 Aug 19;353(6301):790-4. doi: 10.1126/science.aaf7894
10. Head A, Manguin M, Tran N, Blumenstock JE. Can human development be measured with satellite imagery?. Ictd. 2017 Nov 16;17:16-9. doi: 10.1145/3136560.3136576
11. Yeh C, Perez A, Driscoll A, Azzari G, Tang Z, Lobell D, Ermon S, Burke M. Using publicly available satellite imagery and deep learning to understand economic well-being in Africa. Nature communications. 2020 May 22;11(1):2583. doi:10.1038/s41467-020-16185-w
12. Kondmann L, Zhu XX. Measuring changes in poverty with deep learning and satellite imagery. Available from: <https://elib.dlr.de/137108/2/camera_ready.pdf>
13. Hofer M, Sako T, Martinez A, Addawe M, Bulan J, Durante RL, Martillan M. Applying artificial intelligence on satellite imagery to compile granular poverty statistics. ADB Economics Working Paper Series; 2020. doi: 10.22617/WPS200432-2
14. Ledesma C, Garonita OL, Flores LJ, Tingzon I, Dalisay D. Interpretable poverty mapping using social media data, satellite images, and geospatial information. arXiv preprint arXiv:2011.13563. 2020 Nov 27. Available from: <https://arxiv.org/abs/2011.13563>
15. Han S, Ahn D, Park S, Yang J, Lee S, Kim J, Yang H, Park S, Cha M. Learning to score economic development from satellite imagery. InProceedings of the 26th ACM SIGKDD International Conference on Knowledge Discovery & Data Mining 2020 Aug 23 (pp. 2970-2979). doi:10.1145/3394486.3403347
16. Ayush K, Uzkent B, Burke M, Lobell D, Ermon S. Generating interpretable poverty maps using object detection in satellite images. arXiv preprint arXiv:2002.01612. 2020 Feb 5.
    doi:10.48550/arXiv.2002.01612
17. Hall O, Dompae F, Wahab I, Dzanku FM. A review of machine learning and satellite imagery for poverty prediction: Implications for development research and applications. Journal of International Development. 2023 Oct;35(7):1753-68. doi:10.1002/jid.3751
