# Supplementary material for: Shedding light on development: Leveraging the new nightlights data to measure economic progress
Source: PLoS One. 2025 Feb 3;20(2):e0318482. doi: 10.1371/journal.pone.0318482 (PMC11790135; doi:10.1371/journal.pone.0318482)
Supplement: S1 Table — (DOCX) [file pone.0318482.s003.docx]

**S3 Table 1: List of countries and DHS waves in the sample**

| **Country** | **DHS survey years** |
| --- | --- |
| Angola | 2015 |
| Benin | 2012, 2017 |
| Burkina Faso | 2010 |
| Burundi | 2010, 2016 |
| Cameroon | 2004, 2011, 2018 |
| Chad | 2014 |
| Comoros | 2012 |
| Congo Democratic Republic | 2007, 2013 |
| Cote d’Ivoire | 2012 |
| Egypt | 2005, 2008, 2014 |
| Eswatini | 2006 |
| Ethiopia | 2005, 2010, 2016, 2019 |
| Gabon | 2012 |
| Gambia | 2019 |
| Ghana | 2008, 2014 |
| Guinea | 2005, 2012, 2018 |
| Kenya | 2008, 2014 |
| Lesotho | 2004, 2009, 2014 |
| Liberia | 2007, 2013, 2019 |
| Madagascar | 2008 |
| Malawi | 2004, 2010, 2015 |
| Mali | 2006, 2012, 2018 |
| Mozambique | 2011 |
| Namibia | 2006, 2013 |
| Niger | 2012 |
| Nigeria | 2008, 2013, 2018 |
| Rwanda | 2005, 2008, 2010, 2014, 2019 |
| Sierra Leone | 2008, 2013, 2019 |
| South Africa | 2017 |
| Tanzania | 2010, 2015 |
| Togo | 2013 |
| Uganda | 2006, 2011, 2016 |
| Zambia | 2007, 2013, 2018 |
| Zimbabwe | 2005, 2010, 2015 |
