# Supplementary material for: Shedding light on development: Leveraging the new nightlights data to measure economic progress
Source: PLoS One. 2025 Feb 3;20(2):e0318482. doi: 10.1371/journal.pone.0318482 (PMC11790135; doi:10.1371/journal.pone.0318482)
Supplement: S4 Table — (DOCX) [file pone.0318482.s006.docx]

**S3 Table 4: OLS regression for GDP per capita with year fixed effects**

|  | **Gross Domestic Product per capita** | | |
| --- | --- | --- | --- |
|  | **(1)** | **(2)** | **(3)** |
| Nightlights | 0.224*** |  | 0.344*** |
|  | (0.003) |  | (0.003) |
| Population Density |  | 0.062*** | -0.145*** |
|  |  | (0.003) | (0.003) |
| Fixed effects (year) | Yes | Yes | Yes |
| OOS R² | 0.393 | 0.224 | 0.448 |
| Adjusted R² | 0.393 | 0.224 | 0.448 |
| Residual Std. Error | 0.675 | 0.765 | 0.642 |

Notes: Dependent variable is the Gross Domestic Product per capita. Gross Domestic Product per capita, Nightlights, and population density have been transformed using the inverse hyperbolic sine (IHS) transformation. Standard errors (in parentheses) are clustered at the year level. All models have year fixed effects. *p<0.1; **p<0.05; ***p<0.01. Nighttime lights data is derived from Li et al. (2020). Population density is sourced from the GPWv4, and Gross Domestic Product per capita data from Kummu et al. (2018). For more details, see Table 1.
