# Supplementary material for: Shedding light on development: Leveraging the new nightlights data to measure economic progress
Source: PLoS One. 2025 Feb 3;20(2):e0318482. doi: 10.1371/journal.pone.0318482 (PMC11790135; doi:10.1371/journal.pone.0318482)
Supplement: S7 Table — No fixed effects. (DOCX) [file pone.0318482.s009.docx]

**S3 Table 7: OLS regression for HDI. No fixed effects**

|  | **Human Development Index** | | |
| --- | --- | --- | --- |
|  | **(1)** | **(2)** | **(3)** |
| Nightlights | 0.029*** |  | 0.038*** |
|  | (0.0003) |  | (0.0004) |
| Population Density |  | 0.012*** | -0.012*** |
|  |  | (0.0003) | (0.0003) |
| Fixed effects | No | No | No |
| OOS R² | 0.302 | 0.071 | 0.333 |
| Adjusted R² | 0.302 | 0.071 | 0.333 |
| Residual Std. Error | 0.079 | 0.091 | 0.077 |

Notes: Dependent variable is the Human Development Index. Nightlights and Population density have been transformed using the inverse hyperbolic sine (IHS) transformation. Standard errors are in parentheses. These results don’t have fixed effects. *p<0.1; **p<0.05; ***p<0.01. Nighttime lights data is derived from Li et al. (2020). Population density is sourced from the GPWv4, and Human Development Index (HDI) from Kummu et al. (2018). For more details, see Table 1.
