# Supplementary material for: Shedding light on development: Leveraging the new nightlights data to measure economic progress
Source: PLoS One. 2025 Feb 3;20(2):e0318482. doi: 10.1371/journal.pone.0318482 (PMC11790135; doi:10.1371/journal.pone.0318482)
Supplement: S8 Table — (DOCX) [file pone.0318482.s010.docx]

**S3 Table 8: Results from the spatial model with country and year fixed effects.**

|  | **Wealth Index** | | |
| --- | --- | --- | --- |
|  | **(1)** | **(2)** | **(3)** |
| Nightlights | 0.574*** |  | 0.475*** |
|  | (0.005) |  | (0.008) |
| Population Density |  | 0.386*** | 0.105*** |
|  |  | (0.005) | (0.006) |
| Fixed effects (Country and year) | Yes | Yes | Yes |
| OOS R² | 0.478 | 0.351 | 0.489 |
| Adjusted R² | 0.478 | 0.351 | 0.489 |
| Residual Std. Error | 0.699 | 0.872 | 0.684 |

Notes: Dependent variable is the DHS mean wealth index. Nightlights and population density have been transformed using the inverse hyperbolic sine (IHS) transformation. Standard errors are in parentheses. All models have country and year fixed effects and account for spatial auto-correlation. *p<0.1; **p<0.05; ***p<0.01. Household Wealth Index data is derived from DHS. Nighttime lights data is derived from Li et al. (2020). Population density is sourced from the GPWv4. For more details, see Table 1.
