# Supplementary material for: Shedding light on development: Leveraging the new nightlights data to measure economic progress
Source: PLoS One. 2025 Feb 3;20(2):e0318482. doi: 10.1371/journal.pone.0318482 (PMC11790135; doi:10.1371/journal.pone.0318482)
Supplement: Attachment — Submitted filename: Response to Reviewers.docx [file pone.0318482.s011.docx]

**Responses to the comments**

Thank you for the opportunity to revise and resubmit our manuscript “Shedding light on development: Leveraging the new nightlights data to measure economic progress” to PLOS ONE. We sincerely thank the Editor and Reviewers for their insightful and detailed feedback on our manuscript. We deeply appreciate the time and effort dedicated to evaluating our work.

In response to the comments provided, we have carefully revised the manuscript to address all concerns. We believe these changes have significantly enhanced the quality and clarity of the paper.

Below, we provide a detailed point-by-point response to each comment. For ease of review, we have included the original comments in italics, followed by our corresponding responses. Page numbers referenced in our responses correspond to the revised version of the manuscript.

**Responses to Reviewer #1’s comments**:

**Reviewer #1:** *This paper is interesting and contains pertinent information, but it is necessary to review the econometric model because the georeferenced information from neighboring cases can generate the problem of spatial autocorrelation, which limits the validity of the regression coefficients. There is a very rich methodology on econometric models with spatial data. You can consult:*

- *Baltagi, B.H., Li, D. (2004). Prediction in the Panel Data Model with Spatial Correlation. In: Anselin, L., Florax, R.J.G.M., Rey, S.J. (eds) Advances in Spatial Econometrics. Advances in Spatial Science. Springer, Berlin, Heidelberg.* [*https://doi.org/10.1007/978-3-662-05617-2_13*](https://doi.org/10.1007/978-3-662-05617-2_13)
- *Anselin, L., Gallo, J.L., Jayet, H. (2008). Spatial Panel Econometrics. In: Mátyás, L., Sevestre, P. (eds) The Econometrics of Panel Data. Advanced Studies in Theoretical and Applied Econometrics, vol 46. Springer, Berlin, Heidelberg.* [*https://doi.org/10.1007/978-3-540-75892-1_19*](https://doi.org/10.1007/978-3-540-75892-1_19)

Response: Thank you for highlighting this important concern. To address this concern, we conducted Moran’s I tests and Monte Carlo simulations on the residuals of all models in our analysis to determine the presence and significance of spatial autocorrelation. Below are the key findings:

**Moran’s I Tests on Residuals**:

The Moran’s I values for the three models (from Table 3 in main text) are approximately 0.422, 0.501, and 0.422, respectively, with p-values < 2.2e-16. These results indicate a moderate but statistically significant level of positive spatial autocorrelation in the residuals of the three models.

**Table R.1: Moran's I Test Results**

| **Model** | **Moran's I** | **Expectation** | **Variance** | **Std. Dev.** | **p-value** |
| --- | --- | --- | --- | --- | --- |
| Model 1 | 0.42211 | -3.238866e-05 | 1.114922e-05 | 126.43 | < 2.2e-16 |
| Model 2 | 0.50083 | -3.238866e-05 | 1.114914e-05 | 150.0 | < 2.2e-16 |
| Model 3 | 0.42163 | -3.238866e-05 | 1.114918e-05 | 126.28 | < 2.2e-16 |

# Notes: Moran’s I statistic was tested on the residuals of the regression models presented in Table 3. The test assesses the degree of spatial autocorrelation, where a significant result (p-value < 0.05) indicates non-random spatial clustering in the residuals. The weights matrix was constructed using a k-nearest neighbors approach with k=5, and Moran’s I values suggest moderate spatial autocorrelation.

To further investigate the spatial dependency in the residuals, we created Moran's I scatterplots for each model's residuals (Figure R.1). These scatterplots visualize the relationship between residuals and their spatially lagged values. The x-axis represents the residuals from the regression model. The y-axis represents the spatially lagged residuals, calculated using the spatial weights matrix based on k=5 nearest neighbors. A linear fit (red line) was overlaid on the scatterplot to illustrate the trend between the residuals and their spatial lags. A positive linear trend is evident, suggesting that high residuals are geographically clustered with other high residuals, and low residuals are clustered with low residuals.

**Figure R.1: Scatterplots of residuals and their lagged values.**

**Monte Carlo Simulations**:

- To further validate the Moran's I results, we conducted Monte Carlo simulations with 999 permutations for each model. The observed ranks were consistently at the upper bound of the simulations (rank = 1000), with p-values of 0.001. These simulations confirm the robustness of the spatial autocorrelation detected in the residuals.

**Table R.2: Monte Carlo Simulation Results**

| **Model** | **Moran's I** | **Observed Rank** | **Simulations** | **p-value** |
| --- | --- | --- | --- | --- |
| Model 1 | 0.42211 | 1000 | 999 | 0.001 |
| Model 2 | 0.50083 | 1000 | 999 | 0.001 |
| Model 3 | 0.42163 | 1000 | 999 | 0.001 |

Notes: Monte Carlo simulations (999 permutations) were conducted to validate the Moran’s I results for the residuals of the regression models in Table 3. The observed ranks consistently reached the upper bound of the simulations, indicating strong statistical significance (p-value = 0.001). The spatial weights matrix was based on k-nearest neighbors (k=5).

To address this issue, we re-estimated the model using spatial fixed effect models (“spaMM” package in R). Before diving in, we would like to provide some context and clarify our methodological choices. Initially, we aimed to incorporate spatial fixed effect models across the full dataset to account for spatial autocorrelation. Unfortunately, due to the size of the dataset and computational limitations, running these models was not feasible; the process required extensive computational time (up to 72 hours per model) and ultimately resulted in crashing of the software. To address this, we conducted supplementary analyses using spatial econometric models on a subset of the dataset (15,000 observations). This allowed us to evaluate spatial autocorrelation in a more manageable framework. The results of these supplementary spatial econometric models (Table R.3) are consistent with the primary analysis (Table 3 in the main text) and serve as a robustness check. The results of these supplementary spatial econometric models are presented in the supporting information document as S3 Table 8.

**Table R.3: Results from the spatial model with country and year fixed effects.**

|  | **Wealth Index** | | |
| --- | --- | --- | --- |
|  | **(1)** | **(2)** | **(3)** |
| Nightlights | 0.574*** |  | 0.475*** |
|  | (0.005) |  | (0.008) |
| Population Density |  | 0.386*** | 0.105*** |
|  |  | (0.005) | (0.006) |
| Fixed effects (Country and year) | Yes | Yes | Yes |
| OOS R² | 0.478 | 0.351 | 0.489 |
| Adjusted R² | 0.478 | 0.351 | 0.489 |
| Residual Std. Error | 0.699 | 0.872 | 0.684 |

Notes: Dependent variable is the DHS mean wealth index. Nightlights and population density have been transformed using the inverse hyperbolic sine (IHS) transformation. Standard errors are in parentheses. All models have country and year fixed effects and account for spatial auto-correlation. *p<0.1; **p<0.05; ***p<0.01. Household Wealth Index data is derived from DHS. Nighttime lights data is derived from Li et al. (2020). Population density is sourced from the GPWv4. For more details, see Table 1.

Our findings are broadly consistent with those of Baltagi et al. (2004), who demonstrated that accounting for spatial autocorrelation does not always improve prediction accuracy. In their analysis of liquor demand, the fixed effect model without spatial autocorrelation achieved the lowest RMSE (0.1360), closely followed by the random effect model without spatial autocorrelation (0.1367). For short-term forecasts (1 year ahead), incorporating spatial autocorrelation improved performance marginally. However, for longer-term forecasts (2 or more years), the FE and RE estimators without spatial autocorrelation consistently outperformed models that accounted for spatial auto-correlation. Overall, Baltagi et al. (2004) concluded that taking into account heterogeneity across states using FE or RE estimators yielded the best out-of-sample RMSE forecast performance. While spatial autocorrelation provided marginal improvements in the first year, its predictive benefits were minimal beyond that, and the differences between spatial and non-spatial models were not statistically significant.

We have included the results of the supplementary spatial econometric models (S3 Table 8) for the subset of the data to provide additional context and support for our findings. However, the primary models in the manuscript rely on fixed effects without accounting for spatial autocorrelation. We appreciate your understanding of these methodological challenges and believe that the inclusion of supplementary spatial analyses strengthens the validity of our approach. We are happy to provide further details or clarifications if needed.

**Responses to Reviewer #2’s comments**:

**Reviewer #2:** *The document raises a topic that is not innovative: the correlation between light intensity, economic activity and economic growth that translates into economic development. Recently, for example, the institutionalist vision of authors such as Acemoglu & Robinson grants special importance to regulatory frameworks, the development of infrastructure and the formation of quality of life indicators.

Notwithstanding the above, the document has important strengths, which are listed below: 1) a robust, adequate and updated literature review; 2) a clear and consistent wording with the design of a justified and relevant research problem; 3) an interesting delimitation of the object of study, being interested in the countries of sub-Saharan Africa; 4) a robust and innovative methodological proposal with a consistent estimation process; 5) obtaining and analyzing results with a purposeful perspective and contribution to knowledge.*

*The study highlights the relevant evidence regarding the unnecessary correlation of urban lighting presence with population density. Such a result seems to suggest a development dynamic disconnected from quality of life indicators, especially in communities with greater social backwardness in Africa, whose implications may be extensive to different regions of the underdeveloped world.

It is, in summary, a document with all the possibilities of publication, which contributes to the literature, with a well-applied methodology and from which economic policy implications are derived that motivate a reflection on the nature, type and scope of development in regions, which are referents of exclusion in the great processes imposed by globalization.

Finally, although the tabular and graphic schemes are of our own creation, it is suggested that the reader be told the source of the data in order to specify the origin of the information contained in Table 1 in each table and in each graph.*

Response: Thank you for highlighting the need for clearer attribution of data sources in tables and graphs. While Table 1 presents the source of each variable used in our study, we have also revised all tables and figures to include specific data source details and ensure clarity for the audience.

Once again, we appreciate your constructive comments, which have helped us improve the manuscript. We hope the revisions will address your concerns.

**References cited in this note**

Baltagi, B.H., Li, D. (2004). Prediction in the Panel Data Model with Spatial Correlation. In: Anselin, L., Florax, R.J.G.M., Rey, S.J. (eds) Advances in Spatial Econometrics. Advances in Spatial Science. Springer, Berlin, Heidelberg. doi: 10.1007/978-3-662-05617-2_13
